# Length of stay and prior heart failure admission in frailty and heart failure: A systematic review and meta‐analysis

**DOI:** 10.1002/ehf2.15300

**Published:** 2025-04-10

**Authors:** Konstantinos Prokopidis, Amy Nortcliffe, Chukwuma Okoye, Massimo Venturelli, Gregory Y. H. Lip, Masoud Isanejad

**Affiliations:** ^1^ Department of Musculoskeletal and Ageing Science Institute of Life Course and Medical Sciences University of Liverpool Liverpool UK; ^2^ Liverpool Centre for Cardiovascular Science at University of Liverpool Liverpool John Moores University and Liverpool Heart & Chest Hospital Liverpool UK; ^3^ Liverpool Centre for Cardiovascular Science University of Liverpool Liverpool UK; ^4^ Aging Research Center, Department of Neurobiology, Care Sciences and Society (NVS), Karolinska Institutet Stockholm University Solna Sweden; ^5^ Department of Medicine and Surgery University of Milan‐Bicocca Monza Italy; ^6^ Acute Geriatric Unit IRCCS Foundation San Gerardo dei Tintori Monza Monza Italy; ^7^ Department of Neurosciences, Biomedicine and Movement Sciences University of Verona Verona Italy; ^8^ Department of Internal Medicine University of Utah Salt Lake City Utah USA; ^9^ Danish Center for Health Services Research, Department of Clinical Medicine Aalborg University Aalborg Denmark

**Keywords:** Admissions, Frailty, Heart failure, Hospitalization, Length of stay

## Abstract

**Aims:**

The aim of this study was to compare the differences in length of stay (LoS) and prior hospitalization due to heart failure (HHF) in patients with HF and frailty versus without frailty.

**Methods and results:**

From inception until August 2024, PubMed, Scopus, Web of Science and Cochrane Library were searched. To examine the association related to LoS and HHF in patients with HF, a meta‐analysis using a random‐effects model was conducted (CRD42024570604). Our main analysis demonstrated a significantly increased LoS in patients with frailty versus those without frailty [*n* = 10; mean difference (MD): 3.67; 95% CI: 2.26–5.08, *I* ^2^ = 93%, *P* < 0.01]. Likewise, patients with frailty had significantly increased odds of HHF [*n* = 17; odds ratio (OR): 1.76; 95% CI: 1.50–2.07, *I* ^2^ = 81%, *P* < 0.01]. Risk of bias assessment of the included studies was overall fair, while Egger's test showed publication bias regarding studies that examined LoS (*P* = 0.02).

**Conclusions:**

Patients with frailty have longer LoS and more frequent HHF, underscoring the need for early, targeted interventions to manage frailty that may be attributed primarily to ageing and comorbidity‐related status.

## Introduction

Heart failure (HF) is a complex clinical condition characterized by the heart's inability to adequately pump blood, resulting in a number of physiological abnormalities.^1^ It is commonly classified into HF with a reduced (HFrEF) and preserved (HFpEF) ejection fraction of below 40% and above 50%, respectively. Physical frailty emerges as a major concern among the various challenges that patients with HF encounter, which can lead to increased rates of rehospitalization, lower quality of life and mortality.^2^ Physical frailty, defined as a state of increased vulnerability, is a multidimensional syndrome that includes decreased strength, endurance, and overall functional capacity.^3^ Prior hospitalizations due to HF (HHF), which are common during the course of the disease, may contribute significantly to the interplay between HF and frailty, leading to a 1.5‐ to 2‐fold higher risk of all‐cause mortality and hospitalizations.^4^ Hospitalizations may expose patients to prolonged inactivity and consequent deconditioning, affecting long‐term functional outcomes while leading to the development or aggravation of frailty.^5^ Therefore, HHF may be another critical factor that could potentially modify physiological outcomes interconnected with frailty in HF and to the hospital length of stay (LoS).^6^, ^7^ Patients with lower muscle mass and strength are more prone to extended LoS duration,^8^ contributing to acute sarcopenia, a phenomenon that is described by accelerated losses of muscle mass and functional capacity.^9^ Such conditions may raise the risk for recurrent hospitalization, considering the incremental risk for falls, fractures and/or dependency.

Understanding the dynamic link between heart failure, frailty and prior hospitalizations is critical for improving patient care. This link emphasizes the need for comprehensive programmes that address not only the acute manifestations of HF but also the broader implications for frailty, emphasizing preventive measures and targeted therapies to minimize the repercussions of frequent hospitalizations. In this systematic review and meta‐analysis, we attempt to explore (i) the association among heart failure, frailty and LoS and (ii) whether patients with HF and frailty have higher odds of increased HHF compared with patients with HF but without frailty. These findings may help with a more appropriate resource allocation within healthcare systems and the design of preventative strategies through frailty‐tailored interventions in this clinical group.

## Methods

This study was conducted according to the updated 2020 Preferred Reporting Items for Systematic Reviews and Meta‐Analyses (PRISMA) guidelines.^10^ The protocol is registered in the International Prospective Register of Systematic Reviews (PROSPERO) (CRD42024570604).

### Search strategy

Two investigators searched PubMed, Scopus, Web of Science and Cochrane Library from inception until August 2024. The search terms used included “heart failure AND frail*”.

### Inclusion and exclusion criteria

The following criteria were applied for studies to be included: (i) cross‐sectional data from observational studies; (ii) patients with heart failure irrespective of ejection fraction aged ≥18 years; and (iii) studies including data for both patients with and without frailty. Articles were excluded if they: (i) used identical populations as another more recent study that deemed eligible for inclusion; (ii) were reviews, in vivo or in vitro experiments, commentaries or posters; and (iii) were not published as a full text and in English.

### Data extraction

Two investigators independently extracted data from each study, including the name of first author, year of publication, country of origin, study design, definition of frailty, patient characteristics [sample size, age, sex, body mass index (BMI), left ventricular ejection fraction rate (LVEF%), reported comorbidities and outcomes of interest]. Disagreements between authors were resolved by a third investigator.

### Risk of bias

The quality of the included studies was evaluated using the National Institutes of Health (NIH) Risk of Bias Assessment for Cross‐sectional Studies Tool and was performed by two independent reviewers.

### Statistical analysis

Changes in outcomes from patients with HF and frailty and patients with HF but no frailty were compared between groups to calculate the mean differences (MDs) for the evaluation of changes in relation to hospital LoS and the odds ratio (OR) for the odds risk of increased HHF. When studies reported interquartile ranges (IQR), the formula ‘standard deviation (SD) = width of IQR/1.35’ was used to estimate missing SDs.^11^ Statistical significance was evaluated using the random‐effects model and inverse‐variance method.

Statistical heterogeneity of outcome measurements across studies was measured using the overlap of their confidence intervals (95% CI) and expressed as Cochran's Q (Chi‐square test) and *I* ^2^ measurements. Low heterogeneity was defined as *I* ^2^ between 30% and 49%, moderate heterogeneity between 50% and 74%, and high heterogeneity between 75% and above.^12^ In case of high heterogeneity, meta‐regressions were performed using a random‐effects model^13^ based on BMI, LVEF rate, age, and sex, using R software. Given a sufficient number of studies, subgroup analysis according to different definitions of frailty was also performed. Sensitivity analyses were carried out based on variations in reported comorbidities (i.e., patients with frailty had more comorbidities than those without frailty), as well as studies with a high risk of bias. Review Manager (RevMan 5.4.1) software was used to synthesize the meta‐analysis. *P* values less than 0.05 were considered statistically significant.

## Results

**Figure 1:**
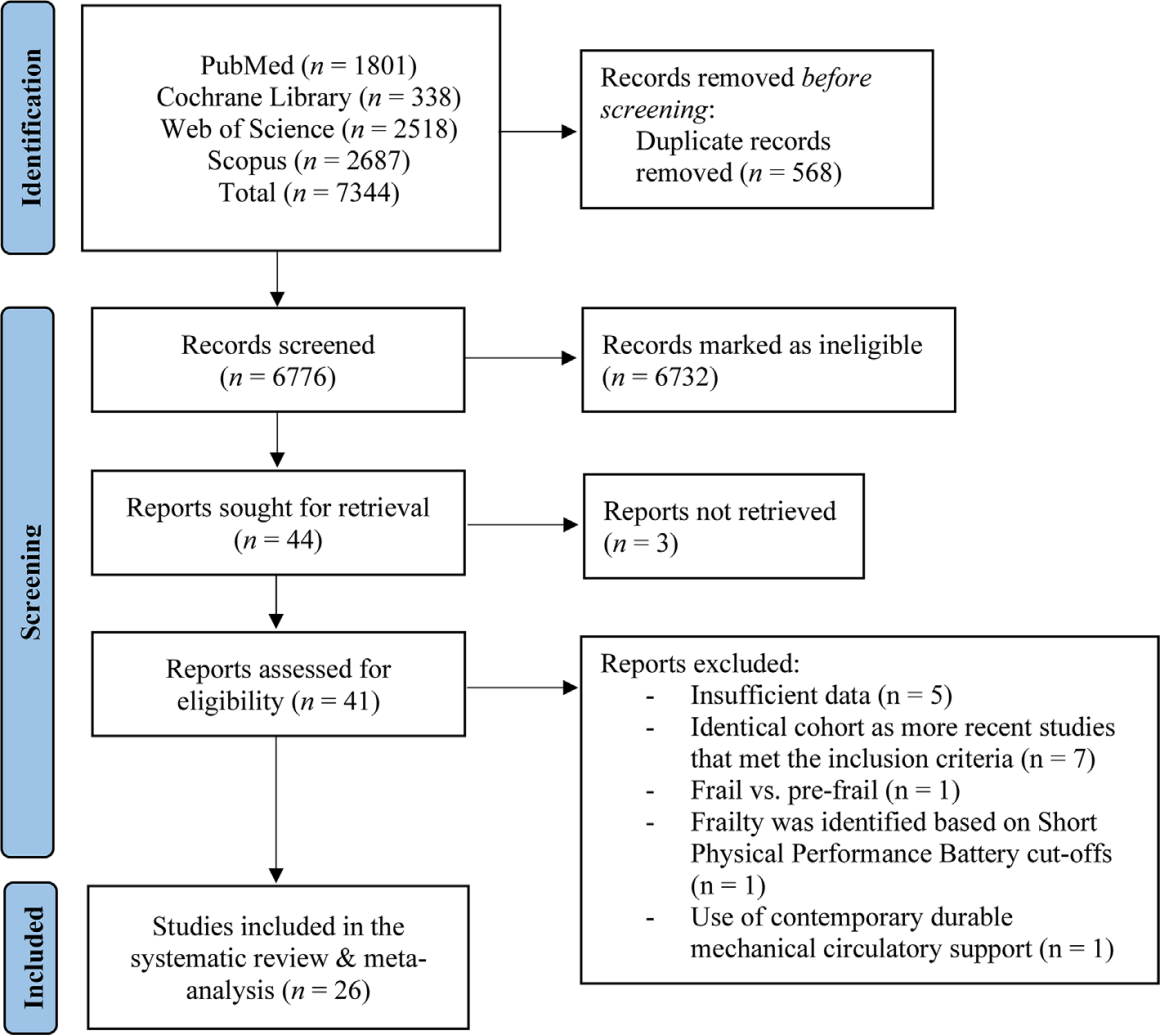
Flowchart of the employed literature search.

The initial literature search provided 7,344 publications. Following the exclusion of duplicates, abstracts, non‐English written text, and studies that full text could not be obtained, 41 full texts were identified as potentially eligible. Of these 41 studies, seven had identical cohort as more recent studies that met the inclusion criteria, five studies had insufficient data, one compared frail versus pre‐frail, one identified frailty based on Short Physical Performance Battery cut‐offs, and in another one, the use of contemporary durable mechanical circulatory support was implicated. In total, 26 studies were included in this systematic review and meta‐analysis (*Figure* *1*). Ten studies examined the differences in LoS,^14^, ^15^, ^16^, ^17^, ^18^, ^19^, ^20^, ^21^, ^22^, ^23^ and 17 studies the odds of prior HHF^17^, ^20^, ^24^, ^25^, ^26^, ^27^, ^28^, ^29^, ^30^, ^31^, ^32^, ^33^, ^34^, ^35^, ^36^, ^37^, ^38^ within the last 12 months. Characteristics of the included studies are shown in *Tables* *1* and *2*.

**Table 1:** Study and participant characteristics of the included studies assessing frailty and length of stay.

Study, year	Country	Frailty Definition	Total *n*	Frail				Non‐frail				Reported comorbidity status
				*n* (M/F)	Age	BMI	LVEF%	*n* (M/F)	Age	BMI	LVEF%	
Ajibawo et al. 2022	USA	Clinical Frailty Scale	1,021	604 (362/242)	70.5 (66.2–71.8)	‐	15 (10–24)	417 (288/129)	70.5 (66.0–74.0)	‐	15 (10–20)	Similar
Su et al. 2024	USA	Hospital Frailty Risk Score	8,495	534 (26/273)	77.2 (11)	27.9 (9.2)	‐	7,961 (4,545/3416)	69.7 (14.1)	29.3 (8.5)	‐	Frail had more AF, diabetes, cerebrovascular disease, MI, DLP, acute kidney injury, CKD, dementia and sepsis
Oguri et al. 2023	Japan	Clinical Frailty Scale	1,053	396 (161/235)	86 (81–90)	21.7 (4.2)	71.7	657 (304/353)	84 (80–88)	21.9 (3.7)	71.2	Frail had more DLP
Lai et al. 2024	Taiwan	Multidimensional frailty index	20,399	8,068 (4,125/3943)	82.4 (7.9)	‐	‐	12,331 (5,777/6554)	78.7 (8.7)	‐	‐	Higher multimorbidity frailty index overall
Kojima et al. 2024	Japan	Clinical Frailty Scale	249	46 (22/24)	84 (78.8–89.3)	23.1 (20.3–25.8)	‐	203 (141/72)	77 (71–82)	23.2 (20.6–26.1)	‐	Non‐frail more HT and DLP; frail had more stroke and dementia
Sharma et al. 2022	Australia	Hospital Frailty Risk Score (HFRS)	109	68 (40/28)	82.4 ± 9.6	‐	‐	41 (25/16)	75.4 ± 14.3	‐	‐	Similar
Hamada et al. 2021	Japan	Kihon Checklist	99	51 (29/22)	84 (77–88)	20.4 (17.9–22.6)	49 (35–63)	48 (23/25)	72 (65–81)	22.3 (20.0–24.4)	46 (32–60)	Frail had more CVA, AF, CKD, malignancy and dementia
Yamada et al. 2021	Japan	Fried (Quartile I vs. IV)	102	17 (11/6)	83 (77–87)	20.9 (18.5–23.5)	‐	85 (60/25)	66 (57–74)	23.1 (20.7–26.0)	‐	Frail had a higher number of comorbidities
Macdonald et al. 2020	Australia	Fried	115	49 (16/33)	51 ± 14	26 ± 6	31 ± 18	66 (43/23)	54 ± 13	26 ± 5	26 ± 13	Similar
Joseph et al. 2017	USA	Fried	489	130 (80/50)	55.2 ± 12.6	29.3 ± 5.1	18.7 ± 10.5	359 (274/85)	62.1 ± 9.7	27.7 ± 6.4	19.2 ± 9.5	Frail had more liver disease

Data are expressed as mean (standard deviation) or median (IQR).

AF, atrial fibrillation; BMI, body mass index; CKD, chronic kidney disease; CAD, coronary artery disease; COPD, chronic obstructive pulmonary disease; DLP, dyslipidaemia; F, females; HT, hypertension; LVEF, left ventricular ejection fraction; M, male; MI, myocardial infarction; PAD, peripheral artery disease, PVD, peripheral vascular disease; T2D, type 2 diabetes; VHD, valvular heart disease.

**Table 2:** Study and participant characteristics of the included studies assessing frailty and previous admission due to heart failure.

Study, year	Country	Frailty Definition	Total *n*	Frail				Non‐frail				Reported comorbidity status
				*n* (M/F)	Age	BMI	LVEF%	*n* (M/F)	Age	BMI	LVEF%	
Kondo et al. 2023	Japan	Fried	542	171 (107/64)	81 (74–86)	20.5 (18.4–22.9)	31.0 (25.2–35.0)	371 (308/63)	61 (51–69)	23.5 (21.1–26.9)	28.0 (23.0–33.0)	Frail had more T2D, and non‐frail had more COPD
Oguri et al. 2023	Japan	Clinical Frailty Scale	1,053	396 (161/235)	86 (81–90)	21.7 (4.2)	71.7	657 (304/353)	84 (80–88)	21.9 (3.7)	71.2	Frail had more DLP
Coats et al. 2023	Worldwide	Rockwood (modified)	2,387	873 (387/486)	71.9 (9.4)	32.7 (6.1)	54.6 (8.7)	1,514 (915/599)	70.7 (10)	27.9 (5.2)	54.4 (9)	Frail had more AF, CAD, diabetes, HT, MI, COPD and stroke
Mizuguchi et al. 2024 (Derivation cohort)	Japan	Clinical Frailty Scale	112	4 (0/4)	86.8 (5)	23.1 (21.9–28)	71 (68–75)	108 (50/58)	81.4 (4.5)	23.1 (20.8–25.1)	60 (43–67)	‐
Mizuguchi et al. 2024 (Validation cohort)	Japan	Clinical Frailty Scale	124	5 (3/2)	89.8 (5.8)	20.3 (18.5–21.8)	57 (57–61)	119 (80/39)	81.2 (4.6)	22.3 (20.3–25.1)	55 (43–65)	‐
Rodriguez‐Pascual et al. 2017	Spain	Fried	497	286 (93/193)	87.5 ± 5.1	‐	‐	211 (101/110)	84.4 (9.4)	‐	‐	Similar
Tanaka et al. 2021	Japan	Fried	418	216 (117/99)	79 (73–85)	22.0 ± 4.5	42 ± 18	202 (120/82)	76 (70–82)	22.5 ± 3.9	43 ± 16	Frail had more AF
Mollar et al. 2022	Spain	Fried	182	121 (58/63)	76 ± 10	‐	49 ± 15	61 (42/19)	70 ± 12	‐	44 ± 15	Similar
Nozaki et al. 2020	Japan	Fried	387	207 (118/89)	76.6 ± 6.1	20.9 ± 3.3	47.4 ± 15.9	180 (128/52)	73.1 ± 5.6	22.3 ± 3.0	45.8 ± 15.7	Frail had more T2D
Vidan et al. 2016	Spain	Fried	416	316 (139/177)	80.76 ± 6.0	27.1 ± 5.6	‐	100 (71/29)	77.87 ± 5.6	26.9 ± 5.1	‐	Similar
Pandey et al. 2022	Multicenter	Rockwood	2,130	1,266 (942/324)	58.9 ± 12.3	31.8 ± 7.3	25.0 ± 7.4	864 (589/275)	58.1 ± 13.3	29.9 ± 6.8	25.3 ± 7.7	Frail had a higher number of comorbidities
Dewan et al. 2020	Multicenter	Rockwood	8,495	3,613 (2,731/882)	67.1 ± 10.3	29.1 (25.7–33.0)	‐	4,882 (3,989/893)	61.0 ± 11.7	26.4 (23.7–29.6)	‐	Frail had a higher number of comorbidities
Sanders et al. 2018	Multicenter	Rockwood	709	227 (119/108)	69 ± 9	37.8 (33.6–43.7)	58 (55–62)	482 (268/214)	73 ± 10	29.4 (26.1–34.4)	58 (50–65)	Frail had more MI, HT and T2D
Butt et al. 2022	Multicenter	Rockwood	3,845	1,491 (841/650)	72.7 ± 8.8	32.1 ± 6.2	‐	2,354 (1,308/1046)	70.1 ± 10.3	28.1 ± 5.8	‐	Frail had a higher number of comorbidities
Sunaga et al. 2021	Japan	Clinical Frailty Scale	842	406 (142/264)	85 (81–89)	22.9 (20.4–26.8)	60–65	436 (235/201)	79 (74–84)	24.2 (21.6–26.9)	60–65	Non‐frail had more DLP
Matsuda et al. 2021	Japan	Clinical Frailty Scale	106	90 (47/43)	81 (76–85)	22 ± 4	59 ± 14	16 (12/4)	51 (38–65)	23 ± 4	63 ± 10	Frail had a higher number of comorbidities
Aung et al. 2021	Multicenter	ASIAN‐HF Frailty Index	2,641	1,448 (1,094/354)	62.6 ± 13.2	25.5 ± 5.9	30.5 ± 13.0	1,193 (858/335)	59.4 ± 13.0	25.2 ± 5.7	33.8 ± 15.0	Frail had a higher number of comorbidities
Hamada et al. 2021	Japan	Kihon Checklist	656	510 (237/273)	84 (77–88)	20.4 (17.9–22.6)	49 (35–63)	146 (83/63)	72 (65–81)	22.3 (20.0–24.4)	46 (32–60)	Frail had more CVA, CKD, AF, malignancy, dementia

Data are expressed as mean (standard deviation) or median (IQR).

AF, atrial fibrillation; BMI, body mass index; CKD, chronic kidney disease; CAD, coronary artery disease; COPD, chronic obstructive pulmonary disease; DLP, dyslipidaemia; F, females; HT, hypertension; LVEF, left ventricular ejection fraction; M, male; MI, myocardial infarction; PAD, peripheral artery disease, PVD, peripheral vascular disease; T2D, type 2 diabetes; VHD, valvular heart disease.

### Length of stay of patients with heart failure and frailty compared with non‐frailty

**Figure 2:**
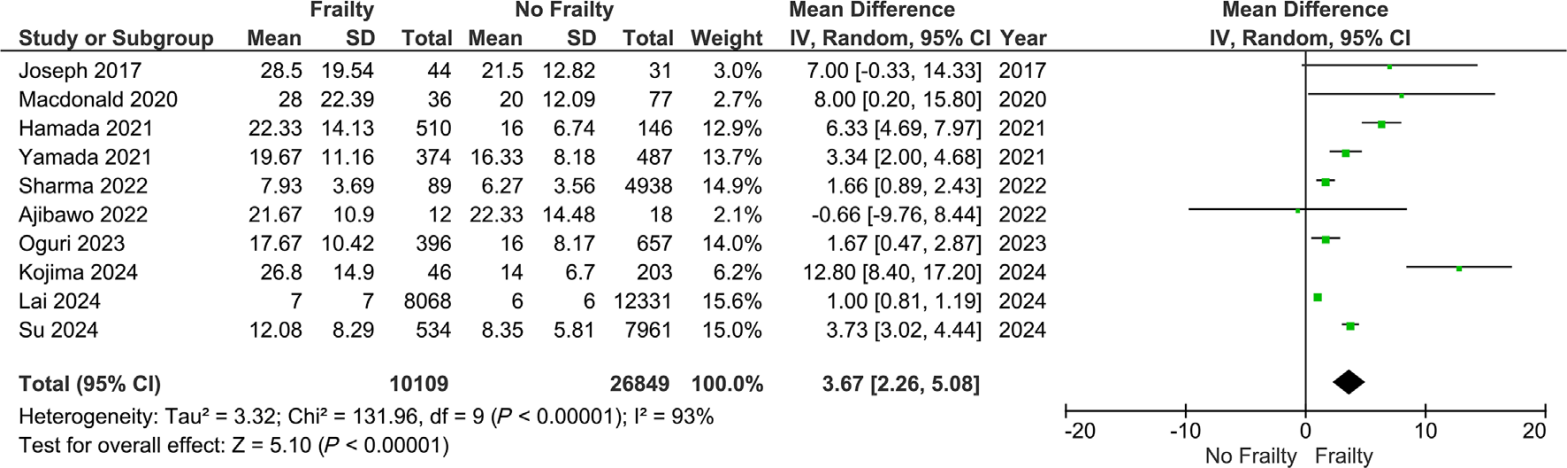
Mean difference of length of stay between patients with heart failure and frailty versus patients with heart failure without frailty.

Our main analysis showed a significantly increased LoS in patients with frailty (*n* = 10; MD: 3.67; 95% CI: 2.26–5.08, *I* ^2^ = 93%, *P* < 0.01) (*Figure* *2*). Sensitivity analysis based on studies with lower risk of bias did not alter the main results (*n* = 8; MD: 2.78; 95% CI: 1.43–4.13; *I* ^2^ = 94%, *P* < 0.01) (*Figure* S1). Considering that most of the studies, patients with frailty exhibited increased prevalence of comorbidities compared with those without frailty, a sensitivity analysis demonstrated no differences in LoS when reported comorbidities were similar between groups (*n* = 2; MD: 4.00; 95% CI: −4.46 to 12.46, *I* ^2^ = 50%, *P* = 0.35) (*Figure* S2). In addition, given that age may be a contributing factor in regard to increasing frailty status, another sensitivity analysis was performed excluding studies for which, those with frailty had a higher mean/median age, showing a statistically significant outcome (*n* = 3; MD: 5.31; 95% CI: 0.35–10.28, *I* ^2^ = 14%, *P* = 0.04) (*Figure* S3).

### Prior hospitalizations in patients with heart failure and frailty compared with non‐frailty

**Figure 3:**
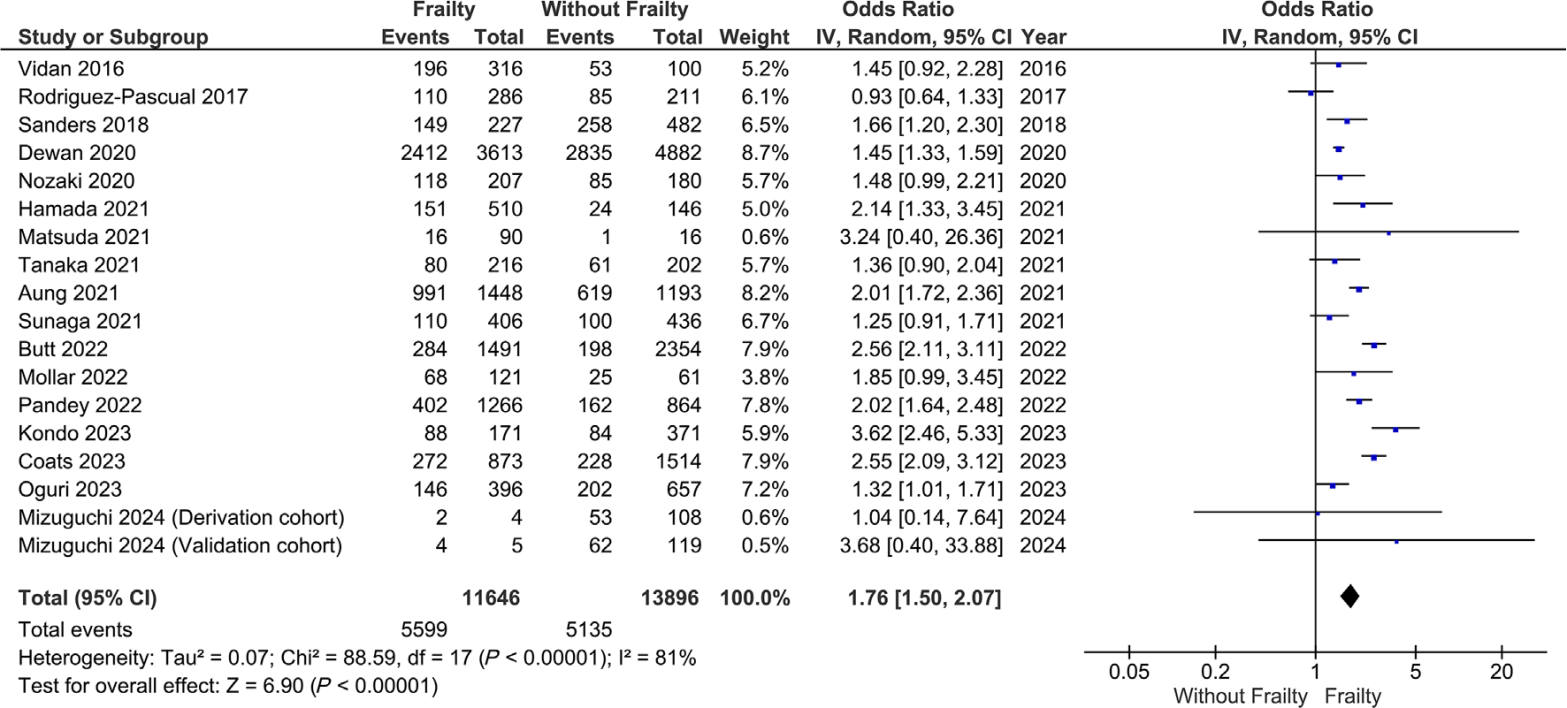
Odds of prior heart failure‐related hospitalization between patients with heart failure and frailty versus patients with heart failure without frailty.

Our main analysis showed that patients with frailty had significantly increased odds of prior HHF compared with those without frailty (*n* = 17; OR: 1.76; 95% CI: 1.50–2.07, *I* ^2^ = 81%, *P* < 0.01) (*Figure* *3*). Subgroup analysis based on different frailty criteria showed that each criterion revealed increased odds of prior HHF (Fried: *n* = 6; OR: 1.61; 95% CI: 1.08–2.42, *I* ^2^ = 81%, *P* = 0.02; Rockwood: *n* = 5; OR: 2.00; 95% CI: 1.51–2.64, *I* ^2^ = 92%, *P* < 0.01; Clinical Frailty Scale: *n* = 4; OR: 1.31; 95% CI: 1.07–1.59, *I* ^2^ = 0%, *P* < 0.01) (*Figure* S4). Sensitivity analysis based on studies for which those with frailty had an increased prevalence of comorbidities versus those without frailty showed no statistically significant differences between groups (*n* = 4; OR: 1.25; 95% CI: 0.97–1.60, *I* ^2^ = 33%, *P* = 0.09) (*Figure* S5). Similarly, a sensitivity analysis based on patients with frailty and significantly higher age showed no statistically significant changes in odds of prior HHF (*n* = 3; OR: 1.28; 95% CI: 0.91–1.82, *I* ^2^ = 64%, *P* = 0.16) (*Figure* S6).

### Risk of bias, publication bias and meta‐regressions

The overall risk of bias assessment of the included studies was overall fair, with two studies having a poor total score (*Table* S1). Egger's test revealed publication bias regarding studies that examined LoS (*P* = 0.02), however, no publication bias was detected in relation to studies examining prior HHF (*P* = 0.19) (*Table* S2). Meta‐regression analyses did not show a moderating effect of age, BMI, LVEF, or sex (proportion of females) to account for the observed heterogeneity. It is worth noting, however, that LVEF and BMI included less than 10 studies to conduct meta‐regressions for studies examining LoS (*Table* S3).

## Discussion

This systematic review assessed the relationship between LoS and prior HHF in HF with and without frailty. Our analysis indicates that patients living with frailty hospitalized for acute HF may be more prone in experiencing longer LoS than those without frailty. In addition, those with frailty also have substantially higher odds of prior HHF compared with their non‐frail counterparts. Moreover, these findings highlights frailty as a potentially risk factor in HF management, underlining its substantial influence on hospital admissions and overall patient outcomes.

HF is a major global cause of hospitalization, predominantly affecting older adults, with four out of five patients being over the age of 65.^1^ Increasingly, there is a shift towards integrating frailty assessments into prognostic and treatment frameworks for HF, aiming to enhance the comprehensiveness of patient management. Frailty represents a clinically observable condition characterized by reduced physiological reserve and higher susceptibility to a wide spectrum of adverse health outcomes.^39^ Frailty frequently coexists with HF, as both conditions arise from shared pathophysiological mechanisms, including a high burden of comorbidities, the effects of ageing, and recurrent hospitalizations, all of which contribute to accelerated functional decline.^4^

The studies included in our review employed various standardized tools for frailty assessment; notwithstanding, frailty assessment can be summarized in two principles: the assessment of frailty as a syndrome or as a state of accumulated comorbidities, being both effective in predicting outcomes in the elderly.^39^ According to our results, the forest plot including HF with comorbidities, in particular, exhibited the strongest correlation, suggesting that this approach may be especially effective in capturing the close relationship between frailty and HF‐related outcomes. Yet, our subgroup analyses reinforced the association between prior HHF and frailty, as detected by different frailty criteria. Our data further reveal an association between increasing levels of frailty and a higher frequency of hospitalizations across the entire spectrum of ejection fraction. Coats^37^ and Sanders^26^ demonstrated a rise in prior HHF among pre‐frail and frail patients with HFpEF using a 44‐item frailty index. These observations are consistent with findings from the PARADIGM and ATMOSPHERE trials, which reported a similar trend in prior HHF with escalating frailty levels, as assessed by the cumulative deficit model in patients with HFrEF.^27^, ^33^

Although frailty assessment has proven effective even in younger adults with HF,^40^ age may significantly influence the relationship between frailty and HHF. Increasing age among frail patients could explain the higher rates of re‐hospitalization, simply due to a longer timeframe for such events to occur. In a study conducted by Rodriguez‐Pascual et al., involving octogenarians, the age difference between frail and non‐frail patients was minimal (87.5 vs. 84.4 years), resulting in no significant difference in prior HHF rates.^25^ Of note, frailty was dichotomized using the Fried criteria (≥3 criteria), potentially misclassifying pre‐frail patients (with 2 criteria) as non‐frail. Similarly, studies by Nozaki et al. and Vidan et al., where the mean age difference was around 3 years, showed an 8%–10% difference in re‐hospitalization among frail and non‐frail patients that did not reach statistical significance.^24^, ^28^ In our sub‐analysis accounting for age, the association between frailty and HHF was attenuated and did not reach statistical significance (OR: 1.28; 95% CI: 0.91–1.82; *I* ^2^ = 64%; *P* = 0.16). This finding highlights the potential for age‐related factors to confound the relationship between frailty and HF outcomes, as older age is inherently associated with both increased frailty and a higher likelihood of HF‐related hospitalizations.

During hospitalization, patients are often subjected to prolonged bed rest and a range of complications, with frailty emerging as a significant both as a risk factor for increased LoS and as a consequence. Moreover, it is estimated that 60% of older patients hospitalized for acute HF present are accompanied by at least one geriatric syndrome.^41^ According to our data, patients living with frailty hospitalized for acute HF have on average 3 days LoS longer than those living without frailty, regardless of age. However, in the sensitivity analysis on studies with similar comorbidity burden, the difference on LoS was no more significant. Notably, the study involved were only two with moderate heterogeneity. This implies that multimorbidity may have a role on the relationship between frailty and LoS; yet, further studies are needed to confirm these assumptions, to tailor preventative interventions on older patients with HF.

These findings are consistent with previous studies that identify frailty as a significant predictor of adverse outcomes in HF. Targeted interventions for frail HF patients should therefore incorporate a comprehensive view of the patient's clinical profile, including comorbid conditions and age‐related vulnerabilities, to enhance care and reduce the risk of HF‐related hospitalizations. In addition, immobilization associated with prolonged bed rest during hospitalization may exacerbate frailty through accelerating losses of skeletal muscle mass, contributing to the risk of hospital‐associated disability syndrome (HADS), sarcopenia, and subsequent disability.^42^, ^43^

Currently, hospital stays often lack early rehabilitation programmes, which could aggravate these risks. While hospitalization in older patients with HF should be avoided when possible, favouring home‐based services,^44^ it remains essential that when hospitalization is necessary, early rehabilitation is imperative during hospital stay. Evidence suggests that such interventions are particularly effective in the most frail HF patients, assisting in limiting the risk of HADS, functional decline, and motor disability.^45^ Recently, a 3‐month multidomain physical rehabilitation intervention that improved quality of life and physical function in patients with HF, demonstrated a much more substantial improvement in those with worse frailty status.^46^ Hence, implementing early rehabilitation in this context could markedly improve patient outcomes such as functional capacity, reduce long‐term disability and enhance overall quality of life.^47^

## Strengths and limitations

Our study has several limitations. First, the high level of heterogeneity across the included studies, particularly in the analysis of prior HHF and LoS (*I* ^2^ > 80% in several analyses), complicates the interpretation of our results and may limit the generalizability of the findings. Second, the variability in frailty assessment tools and criteria, such as the use of the Fried phenotype, Rockwood cumulative deficits model, and Clinical Frailty Scale, introduces challenges in consistently defining frailty across studies, potentially affecting the comparability of results. Furthermore, although sensitivity analyses were performed to address confounding factors like comorbidities and age, the limited number of studies available for these analyses reduces the robustness of these findings, and the absence of statistically significant differences in some subgroups suggests the need for further research. The detection of publication bias in studies examining LoS further suggests that the true impact of frailty on this outcome might be overestimated. Moreover, the reliance on observational studies in this meta‐analysis, which are inherently prone to residual confounding, limits the ability to draw definitive causal inferences between frailty and the examined outcomes. Finally, due to the low number of studies, we could not perform subgroup analyses based on different heart failure phenotypes.

Despite these limitations, our study has several strengths. The use of a comprehensive meta‐analytical approach, including pre‐specified sensitivity analyses, enhances the robustness and reliability of the findings. The inclusion of different frailty criteria allowed for a more nuanced understanding of how frailty is associated with clinical outcomes, thereby increasing the generalizability of our results across different populations. Additionally, the sensitivity analyses performed to account for comorbidities and age differences provided valuable insights into the independent effects of frailty, highlighting the importance of these factors in understanding the clinical trajectory of heart failure patients. The rigorous methodology employed in the risk of bias assessment ensures that the findings are based on studies of overall fair quality. Our study contributes to the growing body of evidence on the impact of frailty on heart failure outcomes, emphasizing the need for integrating frailty assessments into routine clinical practice to better manage the risks associated with heart failure, particularly in older and multimorbid patients.

## Conclusions

Frailty in HF is significantly associated with extended LoS and exhibits a greater likelihood of prior HHF. These results emphasize the profound burden frailty imposes on health care systems and highlight the necessity for developing targeted interventions aimed at managing and potentially mitigating frailty in HF patients, particularly at the initial stages of rehabilitation. Assessment and addressing frailty at admission and improving physical function outcomes could lead to reduced hospitalizations and improved quality of life in HF.

## Supporting information


**Figure S1.** Mean difference of length of stay between patients with heart failure and frailty vs. patients with heart failure without frailty excluding studies with increased risk of bias.

**Figure S2.** Mean difference of length of stay between patients with heart failure and frailty vs. patients with heart failure without frailty based on similar reported comorbidities.

**Figure S3.** Mean difference of length of stay between patients with heart failure and frailty vs. patients with heart failure without frailty based on similar age.

**Figure S4.** Odds of prior heart failure‐related hospitalization between patients with heart failure and frailty vs. patients with heart failure without frailty based on different frailty criteria.

**Figure S5.** Odds of prior heart failure‐related hospitalization between patients with heart failure and frailty vs. patients with heart failure without frailty based on similar reported comorbidities.

**Figure S6.** Odds of previous heart failure‐related hospitalization between patients with heart failure and frailty vs. patients with heart failure without frailty based on similar age.

**Table S1.** Risk of bias assessment of the included studies.

**Table S2.** Egger's test examining potential publication bias.

**Table S3.** Meta‐regression analyses based on age, body mass index, left ventricular ejection fraction, and proportion of females.
