# Supplementary figures and images for: Length of stay and prior heart failure admission in frailty and heart failure: A systematic review and meta‐analysis
Source: ESC Heart Fail. 2025 Apr 10;12(4):2417–26. doi: 10.1002/ehf2.15300 (PMC12287781; doi:10.1002/ehf2.15300)

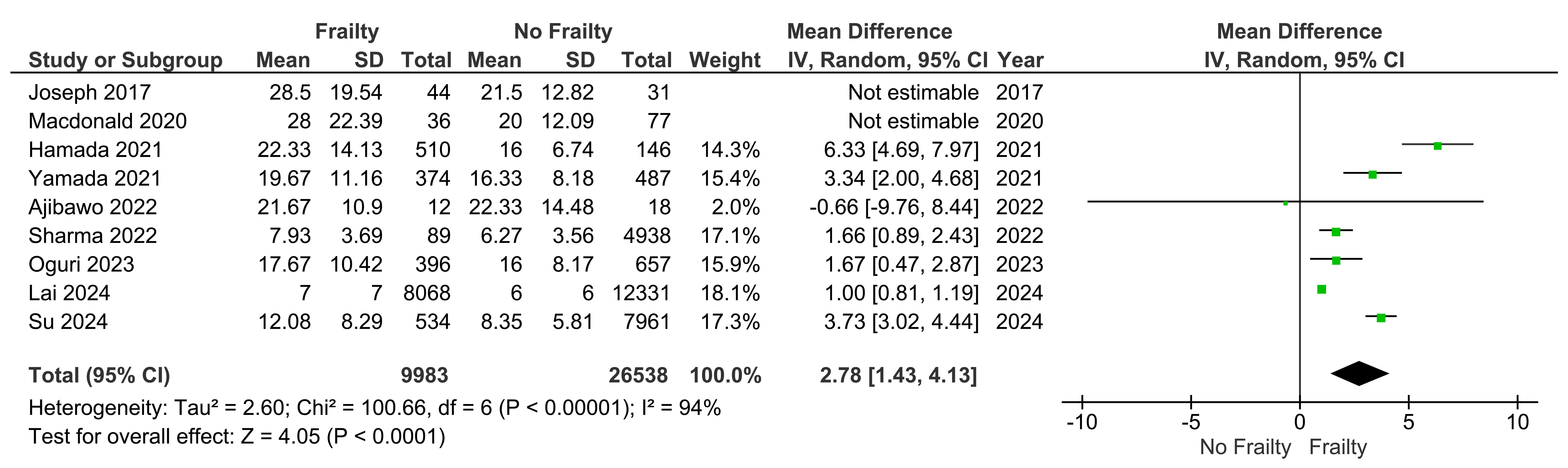

Supplement: Supplementary file 1 — Figure S1. Mean difference of length of stay between patients with heart failure and frailty vs. patients with heart failure without frailty excluding studies with increased risk of bias. [file EHF2-12-2417-s007.tiff]

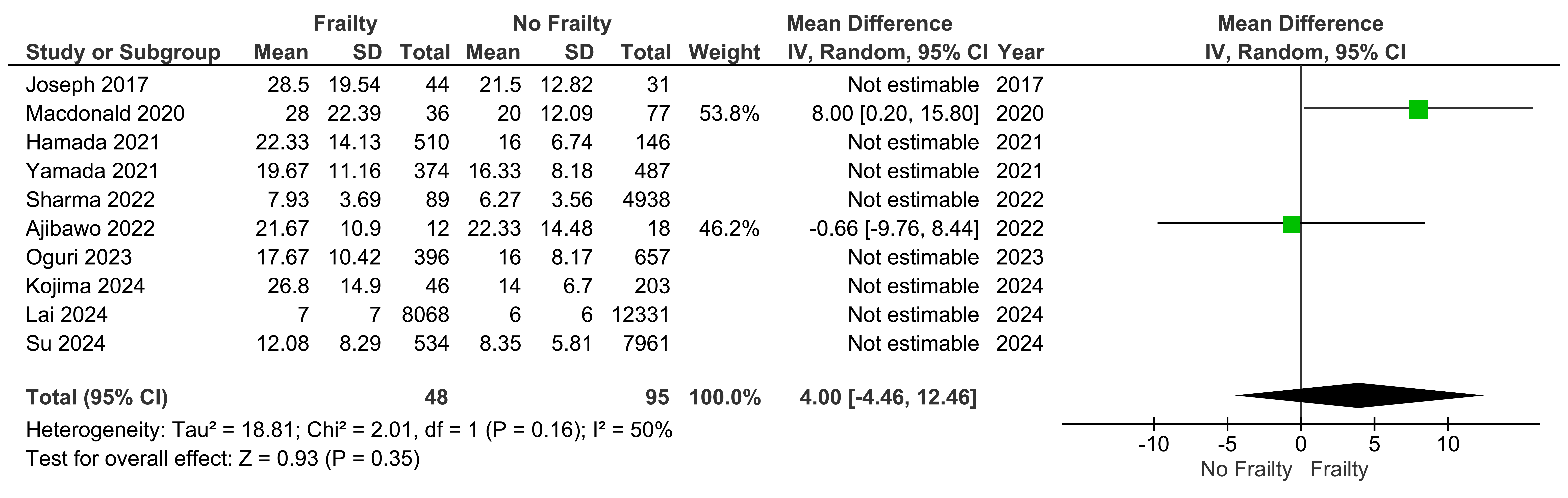

Supplement: Supplementary file 2 — Figure S2. Mean difference of length of stay between patients with heart failure and frailty vs. patients with heart failure without frailty based on similar reported comorbidities. [file EHF2-12-2417-s002.tiff]

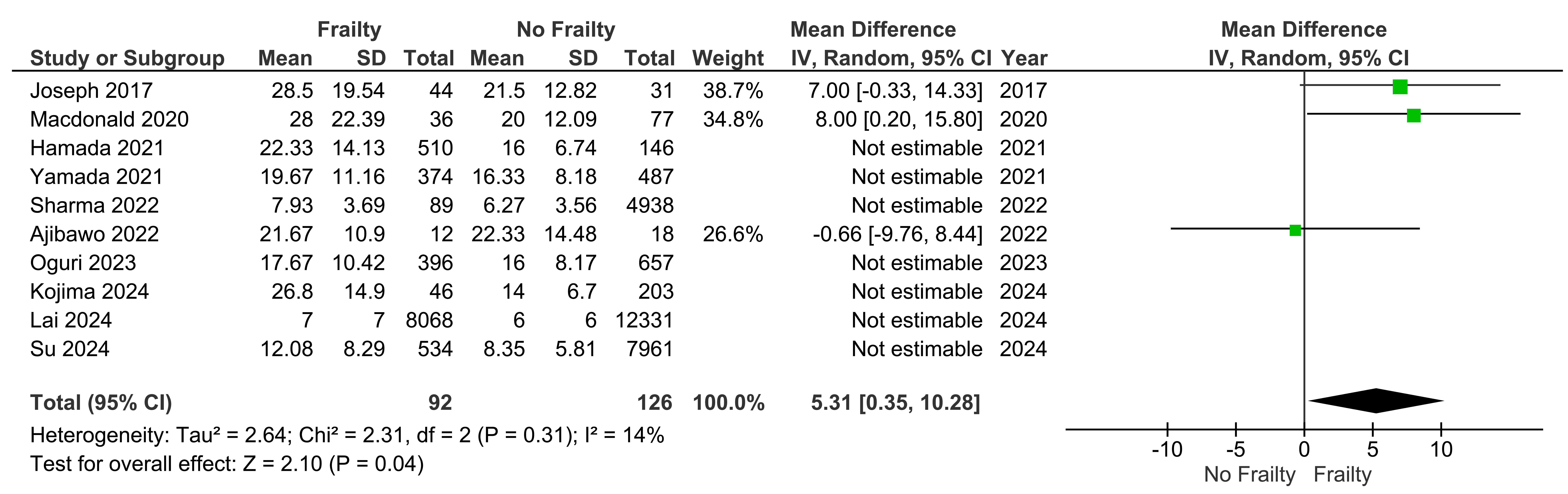

Supplement: Supplementary file 3 — Figure S3. Mean difference of length of stay between patients with heart failure and frailty vs. patients with heart failure without frailty based on similar age. [file EHF2-12-2417-s008.tiff]

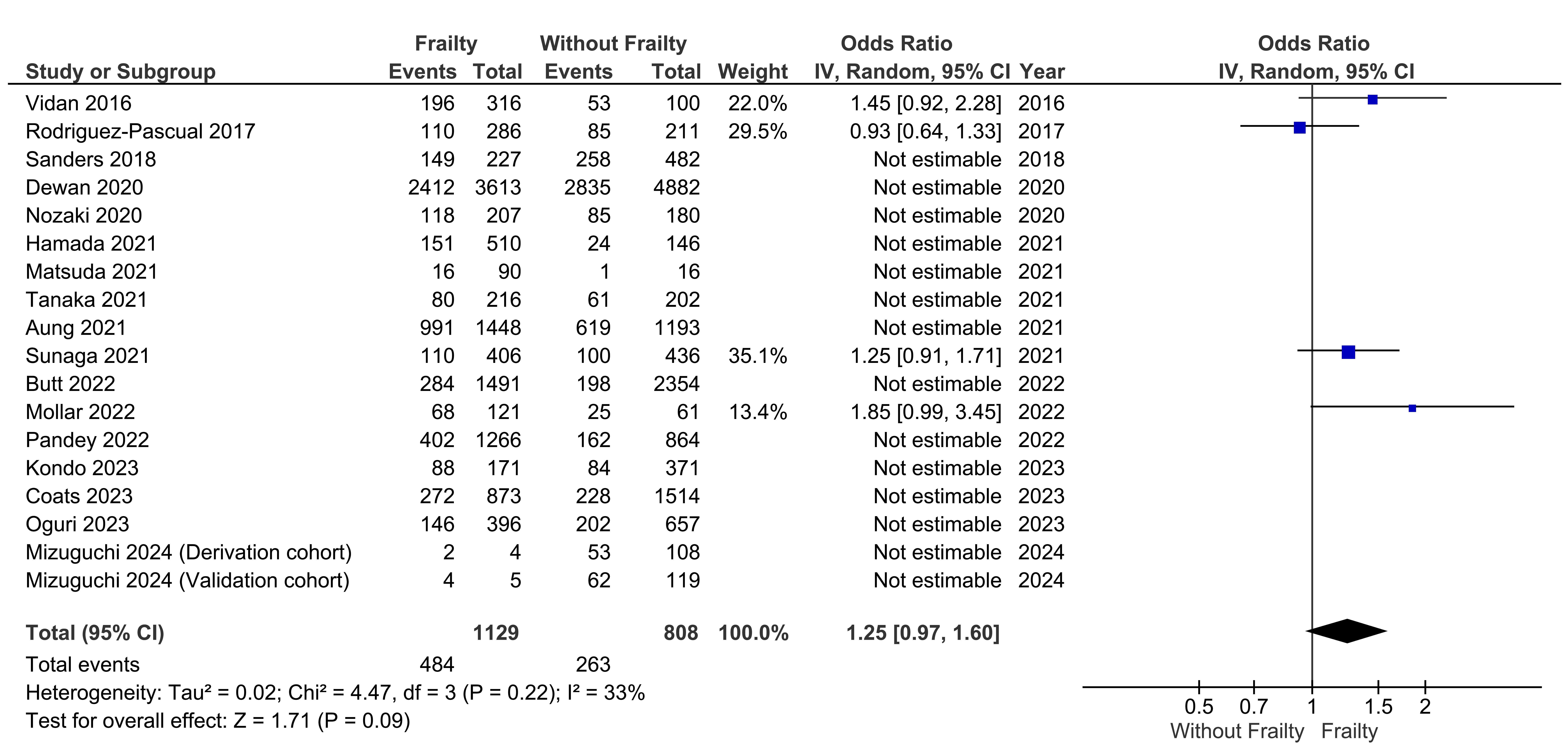

Supplement: Supplementary file 5 — Figure S5. Odds of prior heart failure‐related hospitalization between patients with heart failure and frailty vs. patients with heart failure without frailty based on similar reported comorbidities. [file EHF2-12-2417-s001.tiff]

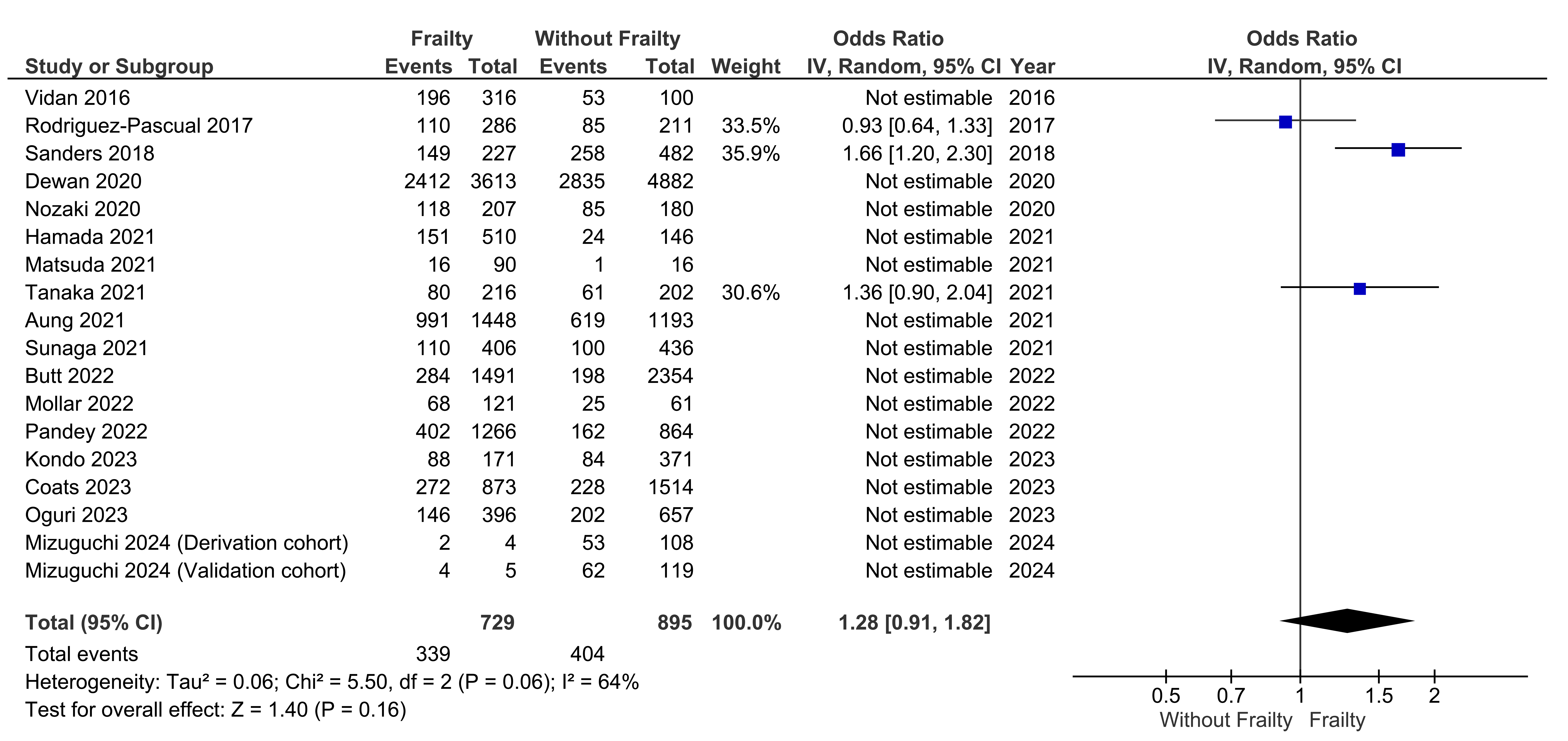

Supplement: Supplementary file 6 — Figure S6. Odds of previous heart failure‐related hospitalization between patients with heart failure and frailty vs. patients with heart failure without frailty based on similar age. [file EHF2-12-2417-s003.tiff]
