# Supplementary material for: Length of stay and prior heart failure admission in frailty and heart failure: A systematic review and meta‐analysis
Source: ESC Heart Fail. 2025 Apr 10;12(4):2417–26. doi: 10.1002/ehf2.15300 (PMC12287781; doi:10.1002/ehf2.15300)
Supplement: Supplementary file 7 — Table S1. Risk of bias assessment of the included studies. [file EHF2-12-2417-s006.docx]

**Table S1.** Risk of bias assessment of the included studies.

| **Study** | **1** | **2** | **3** | **4** | **5** | **6** | **7** | **8** | **9** | **10** | **11** | **12** | **13** | **14** | **Overall Score** |
| --- | --- | --- | --- | --- | --- | --- | --- | --- | --- | --- | --- | --- | --- | --- | --- |
| Kondo 2023 (FLAGSHIP study) | Y | Y | Y | Y | N | Y | Y | Y | Y | N | N | NA (self-reported) | NR | Y | Fair |
| Rodriguez-Pascual 2017 | Y | Y | Y | Y | N | Y | Y | N (frail, not frail) | Y | N | Y | NR | Y | Y | Fair |
| Tanaka 2021 (FRAGILE-HF) | Y | Y | Y | Y | N | Y | Y | Y | Y | N | Y | NR | Y | Y | Good |
| Mollar 2022 | Y | Y | Y | Y | N | Y | Y | Y | Y | N | NR | Y | NR | Y | Fair |
| Nozaki 2020 | Y | Y | Y | Y | N | Y | Y | Y | Y | N | Y | NR | Y | Y | Good |
| Vidan 2016 | Y | Y | Y | Y | N | Y | Y | N | Y | N | Y | NR | Y | Y | Good |
| Pandey 2022 (HF-Action Trial) | Y | Y | NR | N | Y | Y | Y | Y | Y | Y | Y | Y | Y | Y | Good |
| Dewan 2020 (PARADIGM-HF and ATMOSPHERE combined) | Y | Y | Y | Y | Y | Y | N | N | NR | Y | Y | Y | Y | Y | Good |
| Sanders 2018 (TOPCAT trial) | Y | Y | NR | Y | Y | Y | Y | Y | N | Y | Y | Y | Y | Y | Good |
| Butt 2022 (DELIVER)-Trial | Y | Y | NR | Y | Y | Y | N | Y | NR | Y | Y | Y | Y | Y | Good |
| Sunaga 2021 (PURSUIT-HFpEF study) | Y | Y | Y | Y | N | Y | NR | Y | Y | N | N | NR | NR | Y | Fair |
| Matsuda 2021 | Y | Y | Y | Y | N | Y | Y | Y | Y | N | Y | NR | Y | Y | Good |
| Aung 2021 (ASIAN-HF) | Y | Y | Y | Y | N | Y | Y | Y | Y | N | Y | NR | NR | Y | Fair |
| Hamada 2021 (Kochi YOSACOI) | Y | Y | Y | Y | N | Y | N | Y | Y | N | Y | NR | NA – maybe Y since at end of admission (NR) | Y | Fair |
| Ajibawo 2022 | Y | Y | Y | Y | N | Y | Y | N | Y | N | Y | Y | NA | Y | Good |
| Sharma 2022 | Y | Y | Y | Y | Y | Y | Y | Y | Y | N | Y | N | NA | Y | Fair |
| Hamada 2021 | Y | Y | Y | Y | N | Y | N | Y | Y | N | Y | NR | NA – maybe Y since at end of admission (NR) | Y | Fair |
| Yamada 2021 | Y | Y | Y | Y | N | Y | Y | Y | Y | N | Y | NR | NR | Y | Fair |
| Macdonald 2020 | Y | Y | N | Y | N | Y | Y | N (frail, not frail) | Y | N | N (did not detail how outcomes were measured) | NR | NA | Y | Poor |
| Joseph 2017 | Y | Y | Y | Y | N | Y | Y | N (Frail, non frail) | Y | N | N (as above) | NR | Y | N | Poor |
| Su 2024 | Y | Y | Y | Y | N | Y | NA | Y | Y | N | Y | NR | NA | Y | Good |
| Oguri 2023 | Y | Y | Y | Y | N | Y | Y | Y | Y | N | Y | NR | Y | Y | Good |
| Lai 2024 | Y | Y | Y | Y | N | Y | NA | Y | Y | N | Y | NR | NA | Y | Good |
| Coats 2023 | Y | Y | Y | Y | N | Y | Y | Y | Y | N | Y | NR | Y | Y | Good |
| Mizuguchi 2024 | Y | Y | Y | Y | N | Y | Y | Y | Y | N | Y | NR | Y | Y | Good |
| Kojima 2024 | Y | Y | Y | Y | N | Y | Y | Y | Y | N | Y | NR | Y | Y | Good |

1. Was the research question or objective in this paper clearly stated?
2. Was the study population clearly specified and defined?
3. Was the participation rate of eligible persons at least 50%?
4. Were all the subjects selected or recruited from the same or similar populations (including the same time period)? Were inclusion and exclusion criteria for being in the study prespecified and applied uniformly to all participants?
5. Was a sample size justification, power description, or variance and effect estimates provided?
6. 6. For the analyses in this paper, were the exposure(s) of interest measured prior to the outcome(s) being measured?
7. Was the timeframe sufficient so that one could reasonably expect to see an association between exposure and outcome if it existed?
8. For exposures that can vary in amount or level, did the study examine different levels of the exposure as related to the outcome (e.g., categories of exposure, or exposure measured as continuous variable)?
9. Were the exposure measures (independent variables) clearly defined, valid, reliable, and implemented consistently across all study participants?
10. Was the exposure(s) assessed more than once over time?
11. Were the outcome measures (dependent variables) clearly defined, valid, reliable, and implemented consistently across all study participants?
12. Were the outcome assessors blinded to the exposure status of participants?
13. Was loss to follow-up after baseline 20% or less?
14. Were key potential confounding variables measured and adjusted statistically for their impact on the relationship between exposure(s) and outcome(s)?
