# Supplementary material for: Length of stay and prior heart failure admission in frailty and heart failure: A systematic review and meta‐analysis
Source: ESC Heart Fail. 2025 Apr 10;12(4):2417–26. doi: 10.1002/ehf2.15300 (PMC12287781; doi:10.1002/ehf2.15300)
Supplement: Supplementary file 8 — Table S2. Egger's test examining potential publication bias. [file EHF2-12-2417-s005.docx]

**Table S2.** Egger’s test examining potential publication bias.

| **Egger’s test based on studies related to length of stay**  Test for Funnel Plot Asymmetry: t = 2.9856, df = 8, p = 0.0175 Limit Estimate (as sei -> 0): b = 0.7920 (CI: 0.0827, 1.5013) |
| --- |
|  |
| **Egger’s test based on studies related to prior heart failure hospitalizations**  Test for Funnel Plot Asymmetry: t = 1.3568, df = 16, p = 0.1937  Limit Estimate (as sei -> 0): b = 1.4410 (CI: 1.1362, 1.7458) |
