# Supplementary material for: Length of stay and prior heart failure admission in frailty and heart failure: A systematic review and meta‐analysis
Source: ESC Heart Fail. 2025 Apr 10;12(4):2417–26. doi: 10.1002/ehf2.15300 (PMC12287781; doi:10.1002/ehf2.15300)
Supplement: Supplementary file 9 — Table S3. Meta‐regression analyses based on age, body mass index, left ventricular ejection fraction, and proportion of females. [file EHF2-12-2417-s009.docx]

**Table S3.** Meta-regression analyses based on age, body mass index, left ventricular ejection fraction, and proportion of females.

| **Length of Stay** | | | | | | |
| --- | --- | --- | --- | --- | --- | --- |
| Outcome | b | SE | z | p | 95%CI | R^2^ |
| Age | -0.001 | 0.099 | -0.081 | 0.94 | -0.20 – 0.19 | 0% |
| LVEF* | - | - | - | - | - | - |
| BMI* | - | - | - | - | - | - |
| Sex | 0.010 | 0.125 | 0.076 | 0.94 | -0.24 – 0.25 | 0% |
| **Prior Hospitalization due to Heart Failure** | | | | | | |
| Outcome | b | SE | z | p | 95%CI | R^2^ |
| Age | -0.004 | 0.032 | -0.140 | 0.89 | -0.07 – 0.06 | 0% |
| LVEF | -0.022 | 0.047 | -0.475 | 0.63 | -0.11 – 0.07 | 0% |
| BMI | 0.034 | 0.029 | 1.188 | 0.23 | -0.02 – 0.09 | 0% |
| Sex | -0.006 | 0.010 | -0.643 | 0.52 | -0.03 – 0.01 | 0% |

*<10 studies available to conduct meta-regressions
BMI, body mass index; CI, confidence interval; LVEF, left ventricular ejection fraction.
